# A qualitative assessment of older adults’ perspective on the utilization of information from preventive home visits

**DOI:** 10.1007/s40520-025-03069-6

**Published:** 2025-05-20

**Authors:** Anna Nivestam, Ellinor Edfors, Gita Hedin, Albert Westergren, Maria Haak

**Affiliations:** 1 Faculty of Health Sciences, Department of Nursing and Health Sciences, Kristianstad University, 291 88 Kristianstad, Sweden; 2 Department for Research, Development and Education, Management for Psychiatry and Habilitation, Region Skåne, 223 63 Lund, Sweden; 3 Faculty of Health Sciences, Department of Public Health, Kristianstad University, 291 88 Kristianstad, Sweden

**Keywords:** Community planning, Empowerment, Healthy aging, Older adults’ perspective, Preventive home visits

## Abstract

**Background:**

To promote healthy aging, older adults' perspectives should be included in decisions that affect them. Information from preventive home visits, including health data and opinions expressed by older adults, can be used by societal actors to enhance healthy aging. However, information regarding older adults’ perspectives on how this information can be utilized is lacking.

**Aim:**

The purpose of this study was to qualitatively evaluate the perspectives of older adults’ on the utilization of information acquired from preventive home visits.

**Results:**

Older adults view the use of information from preventive home visits as an opportunity to participate in societal decisions and influence changes. This approach can empower older adults by valuing their diverse voices and translating the collected information into meaningful action. The older adults in this study expressed mixed feelings of hope and futility about the possibility of influencing change. They discussed a process for utilizing the information from the visits, which began with highlighting and analyzing problems, communicating these, and then taking action to promote healthy aging.

**Discussion:**

Information gathered during preventive home visits can empower older adults to influence community planning and promote healthy aging. There is a value in involving older adults in decision-making by using their insight to identify barriers and drive change.

**Conclusions:**

Older adults are a valuable resource for community planning aimed at promoting healthy aging. Further research is needed to explore the full potential of using information from preventive home visits for fostering inclusive age-friendly community planning.

## Background

Inclusivity of older adults in decision-making is essential for healthy aging. The World Health Organization [1] plan of action on healthy aging highlights the importance of incorporating older adults’ perspectives in practices and interventions tailored to their needs. While an individual’s preferences, needs, and functional abilities are important considerations, shared decision making is often perceived as valuable and important [2]. However, practical examples of older adults participation in decisions related to areas such as outdoor environments, housing, transportation, and social participation are less reported. Healthy aging is not only fostered through advice and support that maintains a person's physical and mental capacities (intrinsic capacity) but also through an environment that enables older adults to engage in activities they value [3]. Research has suggested evaluating older adults perceptions of their environment and its level of age-friendliness [4]. This could be important for planning, monitoring, and evaluating actions taken in the community [5]. Therefore, considering older adults'perspectives on their daily environments is pivotal for fostering inclusive, age-friendly cities, thereby promoting healthy years of living and independence in later life.

One way to identify the needs and opinions of older adults is to listen to them and include them in political processes [6]. However, some older adults may face barriers to political participation, such as declining health or memory loss [7]. Research has also shown that people with poorer mental and physical health, as well as those with functional impairments and lower incomes, may require environmental adaptations. These accessibility measures, particularly in housing and transportation, may support older adults to function well and to maintain healthy aging [8]. For these and other reasons, incorporating the perspectives of older adults with diverse functional abilities, preferences and needs helps capture the full range of experiences within this population.

Research suggests that preventive home visits can serve as a valuable method of gathering information from older adults, including health data, living conditions, lifestyles, and personal opinions expressed during the visits [9]. Preventive home visits are structured visits typically conducted by healthcare professionals, such as nurses or other healthcare professionals, to assess and support older adults in their home environment. Preventive home visits aim to identify health risks, promote well-being, and facilitate access to necessary resources to enhance healthy ageing. Furthermore, they allow for early detection of health and environmental risks, provide tailored interventions, and support individuals in maintaining their autonomy and well-being [10]. Previous research highlights that home visits can contribute to healthy aging by promoting physical and mental health, improving self-care practices, and facilitating access to community resources [11, 12]. The effectiveness of preventive home visits is debated. For example, a systematic review from 2015 suggests that preventive home visits may contribute to lower mortality rates and improved functional abilities [10]. However, Ramli et al. [13] and Mayo-Wilson et al. [12] indicate that the evidence for significant positive effects is not conclusive.

There are different models of preventive home visits. Overall, preventive home visits offer healthcare professionals the opportunity to probe targeted questions that capture an individual’s experiences within their environment. One such example is about housing preferences, to create environments that support healthy aging. Additionally, decision-makers have suggested that collecting information from older adults during preventive home visits could generate both qualitative insights and statistical data [9], which are not usually obtained from surveys mapping older adults'preferences and living conditions [14, 15].

The information from these visits could also be more detailed at a local level than national surveys, allowing municipalities to have input on the type of information they want from older adults in their specific areas. Therefore, preventive home visits can play a crucial role in informing societal actors about older adults’ preferences and fostering age-friendly planning. It has not previously been studied how older adults view the use of information from home visits for community planning to promote healthy aging. A clearer focus on older adults’ perspectives is crucial to ensuring that community planning truly meets older adults' needs. Therefore, the purpose of this study was to qualitatively evaluate the perspectives of older adults on the utilization of information from preventive home visits for community planning to promote healthy aging. Specific research questions are: How do older adults view the use of information from preventive home visits for community planning that promotes healthy aging? How can this information be effectively used?

## Methods

### Research design

This study used a qualitative design and focus group discussions.  A qualitative approach is well-suited to the aim of the study because it seeks to understand what perspectives older adults attach to the use of information from preventive home visits. The use of focus groups allows to understand context-specific aspects that shape the older adults’ perspective of the phenomenon at target [16, 17]. The essence of focus group discussions is to create new knowledge together among the participants in the group. The participants in the group reason together, building on each other's different perceptions to collectively create knowledge [18].

### Context

This study was conducted as part of the research and collaboration project “Preventive Home Visits to Seniors” [11], which involves eight municipalities in southern Sweden. These municipalities follow a mutually agreed upon model to offer preventive home visits to older adults around 77 years old who are not receiving home care. All municipalities have their own employed home visitors, with varying professions (e.g., nurse, social care worker, assistant nurse), who conduct the visits. The home visitor gives information and advice to the older adult, for example about services offered by the municipality. In addition to this, the home visitor collects information on various aspects of health, well-being, nutrition, social participation, living conditions, home safety, and housing situation. The responses to predetermined questions are recorded in a written format within a digital support system (Entermedic, Entergate, Sweden). Additionally, observations made by the home visitor and other thoughts expressed by the older adults are documented in the digital support system Entermedic. There is an intention, set by the municipalities, to use this collected information for community planning. For example, some municipalities present the information gathered during these visits in political forums.

### Sample, procedure and data collection

To recruit participants eligible for the focus group discussion, purposive sampling was used. After a home visit, five gatekeepers, each representing a different municipality informed presumptive participants about the study and provided them with written information outlining its purpose. With the presumptive participants’ consent, the gatekeepers then shared the names and phone numbers of those interested in participating with the first and second author. The second author then phoned them to offer more detailed information and to schedule the focus group sessions.

The sample was homogeneous in terms of age, consisting of participants residing in ordinary housing without receiving any home help or care services from the municipalities. At the same time, the groups were heterogeneous in composition, including participants with different genders, prior work experience and educational background. A total of 27 participants were initially recruited, but 10 later withdrew for various reasons. In the end, 17 participants took part in the study, consisting of 10 women and seven men, aged 76 to 81. They had prior work experience in areas such as healthcare, IT, teaching, construction, transport, and the military. Their educational background ranged from secondary school to master’s degrees.

Data were collected during five focus group discussions held in five different municipalities close to the participants’ homes. The number of participants in each group ranged from two to six. The first author acted as the moderator for the discussions, while the second author served as the co-moderator. A set of themes guided the discussions, and these were: identifying information for community planning that promotes healthy aging; understanding how societal actors can benefit from insights gained during the visits; and exploring the possibilities and obstacles of sharing this information with various societal actors. Follow-up questions were asked to encourage reflection and elaboration. For example: *Can you explain more? What do you others think about that?*

The discussions lasted for 1.5–2 h. Directly after each focus group the moderator and co-moderator reflected on key themes, insights and concerns that emerged during the focus group discussion. Each focus group discussion was audio recorded and then transcribed verbatim using Amberscript, a transcription software. Thereafter, the first author listened to all audio files and corrected for transcription errors. Quotes used in the manuscript were then translated by the first author with the assistance of the artificial intelligence tool Copilot. To ensure the original meaning of the quotes the translations were then reviewed by all co-authors.

### Data analysis

The focus group discussions were analyzed using Krueger’s framework [16, 17]. While each focus group had its own dynamics, the findings across the groups were largely consistent. Minor differences in perspectives were noted but did not alter the overarching categories. These differences were not clearly attributable to the specific municipality. Reflective notes taken after each session highlighted key points. The first and second authors thoroughly reviewed the material and independently identified themes, starting with data from one focus group, which they then compared and discussed. After the initial review, the first author systematized all discussions within each focus group into themes. The first and second authors discussed the content of each category and wrote a summary representing each category. The summary of each category was shared with the third, fourth, and fifth authors, who verified them against the audio recordings. A preliminary results summary of the two emerging categories was presented during a member check [19]. A written version of the preliminary results was shared with the participants prior to the member check. Individual member checks were conducted with 11 participants through phone calls made by the second author to collect their reflections. Three participants joined an online seminar to share their feedback. All (n = 14) who took part in the member check confirmed and agreed upon the results. During the member check they emphasized the importance of receiving feedback on the actions taken based on the information gathered from the preventive home visits. This emphasis was further reflected in the final results. After the member check, the overarching category was decided in discussions among the authors, highlighting a synthesis of the two categories and a deeper understanding of the results. Finally, all authors reviewed, discussed, and agreed on the results. To strengthen the trustworthiness of the study, we adhered to the principles of the Consolidated Criteria for Reporting Qualitative Research (COREQ) guidelines.

## Results

Older adults’ perspectives on the utilization of information from preventive home visits for community planning to promote healthy aging is illustrated in the overarching category *Potential to participate and influence changes- empowers older adults* and its two sub-categories *Opportunities to influence – mixed feelings of hope and futility* and *Opportunities to highlight barriers and take action* (Table 1). The overarching category *Potential to participate and influence changes- empowers older adults* encapsulates older adults' perspectives on how information from preventive home visits can be utilized for community planning that promotes healthy aging. By utilizing the information from the visits, the older adults' voices can be heard in societal decision-making and potentially influence change. This approach not only empowers older adults by incorporating their diverse voices, but also provides politicians with a basis for informed action. The older adults indicated that using the information from preventive home visits was a legitimate way to get their voices heard. This overarching category covers the two sub-categories *Opportunities to influence – mixed feelings of hope and futility* and *Opportunities to highlight barriers and take action*.

**Table 1:** The overarching category and its sub-categories illustrating older adults' perspectives on the utilization of information from preventive home visits for community planning that promotes healthy aging

Category	Sub-categories
Potential to participate and influence change – empowers older adults	Opportunities to influence – mixed feelings of hope and futility
	Opportunities to highlight barriers and take action

### Opportunities to influence – mixed feelings of hope and futility

The older adults viewed the utilization of information from preventive home visits as offering a chance to influence decision-makers and other societal actors but expressed mixed feelings of hope and futility. First, the older adults shared hopeful feelings about the utilization of information from preventive home visits. They discussed their appreciation of the chance to speak with a person (the home visitor) during the home visit, who could then pass on their information, to actors in society. During the conversation with the home visitor the older adults mentioned that they had time to think about what to convey to decision-makers or other societal actors and reflect on the questions asked by the visitor. The older adults discussed how this differed from other forums, where influencing community decisions often required opinions to be expressed in group settings, which was less secure than doing so in the privacy of a home visit.

The older adults expressed a desire to be invited to engage in dialog with decision-makers to influence societal decisions. They hoped the home visit would be an alternative to such dialog, with the visitor acting as a link between the older adult and the decision-maker. Moreover, the older adults expressed hope that by sharing their opinions, they could contribute to change and thereby influence societal conditions over time. While they acknowledged that not all their wishes could be fulfilled, they still felt that communicating their experiences and preferences provided an important opportunity to be heard. Sharing this information with decision-makers was discussed as a way to advocate for improvements even if only to a modest extent. For example, regarding the type of housing the older adults wanted, or the kind of care desired from the municipality. Thus, there was a feeling of hopefulness that by conveying the information from the visits, their opinions could make a difference to a diverse range of societal actors such as health care, grocery stores and the transport sector. The older adults discussed their hope that the home visitor would incorporate their opinions collected during the visits. They highlighted that the home visitor could use the information in conversations with decision-makers or other community actors to represent the older adults' voices. This was described by the older adults as a good way to make their and other older adults' voices heard when they might not have the energy or find it difficult to participate in political forums. This is illustrated in the following quote from focus group two:

“Actually, it takes a bit of effort and you don't always have that strength. (Participant 1)

No. Can you elaborate on what you mean by that? (Moderator)

And where, where should one turn? And so on. You somewhat stop trying to make your voice heard. (Participant 1)

Yes, because it's not worth it. (Participant 2)

No, you think so. (Moderator)

Yes, you hear politicians and others, they'd rather talk than… actually do something. And maybe it's the same [in this municipality as where the participant lived previously]. Now I have no experience of it here…(Participant 2).

It's not better here. (Participant 1)

You don't think so? (Participant 2)

No. It's not easy to make your voice heard. That effort diminishes. Yes. And then you could get a forum for it. But how? How do you get people to go there? [home visits are described as an alternative] (Participant 1)”.

At the same time, the older adults discussed a feeling of futility about influencing actors in society, especially politicians. The older adults recognized that their opinions expressed during the home visits could not always be considered by decision-makers because they represented just one person's opinion. If several people shared the same opinion, it seemed more legitimate. They were also aware of the economic limitations present in the municipalities, which they felt hindered decision-makers from taking actions according to the suggestions given by the older adults. It was mainly the economic limitations of the municipality and other actors that led to feelings of futility.

The older adults discussed how this sense of futility about influencing and making changes could lead them to give up and refrain from expressing their opinions. They also discussed how they sometimes felt that they were merely a burden, which made them hesitant to express their opinions. The older adults also discussed a sense of futility about influencing decisions; they had a feeling that everything had already been decided. Moreover, they described it as a balancing act; they wanted to express opinions without being perceived as a complaining person. The older adults expressed a fear that if perceived as a complaining person, the information would not be forwarded, and no action would be taken. Furthermore, they said that changes were rarely seen, or feedback was rarely given on what had been conveyed in various forums, leading to little or no trust in decision-makers. When they saw that the desired changes never occurred, trust was lost.

### Opportunities to highlight barriers and take action

The older adults saw the opportunity to systematically highlight barriers and needs using the information from the visits. They believed that passing on this information may enable politicians to take action and plan for the future. The information collected during the home visits could highlight barriers and needs among the older adults. The older adults discussed how, by compiling information from multiple visits, a comprehensive picture of how several people thought and felt was obtained, including for example how many experienced loneliness or had difficulties getting to various activities. They said that they became stronger together when many people had similar thoughts or feelings about actions that could be taken. The view was expressed that more pressure is put on decision-makers when multiple people agree and have the same needs or desires.

When barriers or needs were identified using the information from the visits, the older adults highlighted that these could be analyzed. Additional questions could be asked when the data from the visits indicated a problem. They said that it was important to analyze the problem, why it occurred and what could be done about it. By gathering more opinions and thoughts, responses to what could be done to tackle the problem could be obtained to address the problem or meet the need. Thus, the older adults discussed how the problem could be analyzed, its cause identified, and solutions could be identified. Moreover, the older adults highlighted that when barriers or needs were identified in a municipality, these could be compared with other municipalities. Perhaps the same problem existed in other municipalities, and common solutions could be found. The older adults said that common solutions could lead to increased equity for older adults in different municipalities. Different age groups that had received home visits could also be compared; the older adults discussed whether perhaps the same trend was seen earlier, making the need for action more justified. The importance of comparing data from different municipalities is highlighted in this quote from focus group three:

“No, exactly. They might think the same in XX [another municipality] or YY [yet another nearby municipality], or wherever else. I don't know [if the older adults in other municipalities think the same]… I don't know how troublesome it is over there toward YY [another municipality] direction. (Participant 2).

What do you think? (Moderator)

Money and such. (Participant 2)

Could you add opinions and thoughts on that [from other municipalities]? (Moderator)

Yes, you could bring them together. (Participant 3)

We could see if it's just us who are complaining. (Participant 2)

How it is in other communities [can be compared]. (Participant 3)

[Do] they [older adults in another municipality] also [have the same thoughts about] housing and the food and the communications? (Participant 2)”

The older adults said that when a comprehensive picture was obtained, barriers and needs highlighted, analyzed, and solutions identified, this information could be passed on to decision-makers. The information could be forwarded by the visitor. If the visitor shared the information along with someone else, such as a manager or researcher, the older adults stressed that it helped to increase the legitimacy of the proposals. It was also mentioned by the older adults that decision-makers could use the information from the visits to make immediate decisions and plan for the future. There was an awareness among the older adults that the information collected during the home visits reflected one age group and not all older adults. At the same time, other older adults' opinions were represented by describing those with different needs. Opinions were also expressed about how this group of older adults would like things to be if they became alone or if their health deteriorated. The older adults said that this meant that the information from the visits reflected not only the current needs of those visited but also potential future needs. Finally, the older adults expressed a desire for feedback and visible actions based on the information from the home visits shared with decision-makers.

## Discussion

The present study focused on understanding older adults’ perspectives on utilizing information from preventive home visits for community planning that promotes healthy aging. Our main results showed that by using the information there is potential for older adults to participate in societal decisions and influence societal changes, and thereby empowering them. The older adults said that the use of the information would give them opportunities to influence societal actors, but they had mixed feelings of hope and futility. Using the information could open up opportunities to highlight barriers among older adults and take action to promote healthy aging.

This study emphasizes the important role of older adults in actively contributing to community planning. The results showed that information collected during preventive home visits can be utilized by decision-makers or other societal actors. By utilizing this information, older adults can be empowered to influence community planning. Previous research shows that older adults are given lots of information and recommendations during preventive home visits [20]. Information about, for example, services provided by the municipality, malnutrition and fall risk. Communication occurs from the home visitor to the older adult and the older adult can be seen as a recipient of information [21, 22]. However, the present study shows that information can also go from the older adult to the home visitor. This allows the older adult to have an impact on society, to be an active agent, and not act only as a recipient of information from society. One can visualize this as a mutual exchange between the visitor and the older adult—a bridge linking the older adult and society together.

By utilizing information from preventive home visits, older adults can become empowered. The present results show that utilizing information from preventive home visits can give older adults opportunities to influence community planning and opportunities to highlight barriers and take action that can affect healthy aging. The current results also show that older adults may experience a sense of futility when expressing their opinions and they may hesitate to share their thoughts because they feel like a burden to society. It has been reported that ageism has increased over the years [23]. Previous research indicates that ageism can affect older adults' psychological well-being [24] and, consequently, their ability or willingness to participate in societal discussions. One way to tackle the societal problem of ageism could be to acknowledge older adults as full of potential and capacities and as a productive resource for society instead of a burden. Further participants expressed a sense of limited legitimacy when their input was perceived as representing a single voice. This aligns with previous findings showing that older adults’ perspectives are more likely to be considered when aggregated, for instance through surveys or group-level feedback [5]. While qualitative approaches such as ours offer rich, contextual insights, previous large-scale studies [e.g., 10, 14] demonstrate that combining individual and collective perspectives, such as through surveys, can possibly enhance the perceived legitimacy and usability of older adults’ input. This supports our participants’ reflections that the influence of their perspectives increases when they are echoed by others, as collective voices tend to carry greater weight in decision-making processes. Thus, acknowledging older adults as a resource can improve their quality of life. For example, research shows that engaging older adults in purposeful activities such as helping others can improve their well-being [25]. However, older adults’ productivity in society is often overlooked and undervalued [26]. Listening to older adults' opinions and thoughts and taking them into account in community planning could be a first step toward visualizing older adults as a resource for society. Giving older adults the power to decide about what and how they can contribute in a meaningful way would probably benefit both older adults and society.

### Methodological considerations

Through focus group discussions, a collective understanding was created based on the participants' perspectives [18]. The moderator stimulated the discussions by asking probing questions about the other participants' reflections on the topic discussed. This made the discussions fruitful and lively. Our impression was that the participants found the aim of this study interesting, which contributed to the vibrant discussions.

Five different municipalities were selected, partly because each had different home visitors conducting the visits. This variation provided diverse insights into how information from the visits could be utilized for community planning to promote healthy aging. Municipality-level characteristics were not systematically compared and this may have influenced the results to a limited degree. Neither were individual data on participants’ comorbidities, functional abilities, and cultural backgrounds collected, which can be considered a limitation. Such characteristics may have influenced participants’ views, and future research should explore their potential impact. Further, there were between two and six participants per focus group. The recommended number of participants in each group varies in the literature from four to 12 [18, 27]. We acknowledge having only two participants might be a limitation of this study. However, the aim was not to obtain representative data for the entire municipality, but to gain qualitative insights into how information from preventive home visits can be utilized in community planning. Despite the small number, the discussion was rich and dynamic, with both participants actively engaging in the conversation. Previous literature suggests that smaller focus groups can facilitate deeper exploration of experiences, especially when the topic is personally relevant [16, 17]. The intimate setting may also have fostered a more reflective and open dialogue. Therefore, we consider the data generated from this group to be valuable and consistent with the study’s aim of exploring older adults’ perspectives.

In all groups, everyone was very engaged in the topic and eager to discuss it, indicating that the topic was important to them. This might have been because they felt confident talking to each other, and the atmosphere became almost friendly. They were given the time to develop their discussions and were allowed time to think and reflect. Moreover, we would argue that the results could be transferred to other contexts as well. For example, opinions and thoughts as well as information from older adults could be collected in other forums such as healthcare situations where personnel meet older adults. This information could then be forwarded to societal actors by the personnel.

## Conclusions

This study shows that older adults can be considered as a resource in society by using the information from preventive home visits for community planning that promotes healthy aging. When societal actors consider this information, older adults gain the opportunity to participate and influence meaningful change. It is essential not to overlook the potential for mutual information exchange between societal actors, home visitors and older adults that these visits provide. Those conducting preventive home visits or similar initiatives have to be aware of this opportunity and the additional purposes of the visit. Further research is needed to synthesize the scientific literature and explore how information gathered during preventive home visits can be effectively utilized in community planning to promote healthy aging.

## Data Availability

The datasets generated during and analyzed during the current study are not publicly available for confidentiality reasons but are available from the corresponding author upon reasonable request.
